# Lung Function Decline after 24 Weeks of Moxa Smoke Exposure in Rats

**DOI:** 10.1155/2019/9236742

**Published:** 2019-01-10

**Authors:** Rui He, Li Han, Ping Liu, Hai Hu, Jia Yang, Hong Cai, Chang Huang, Lei Wang, Juntian Liu, Jian Huang, Lue Ha, Yaomeng Liu, Jihong Wu, Maoxiang Zhu, Baixiao Zhao

**Affiliations:** ^1^Beijing University of Chinese Medicine, Beijing, China; ^2^Beijing Electric Power Hospital, Beijing, China; ^3^Beijing Hospital of Acupuncture and Moxibustion, Beijing, China; ^4^Chongqing Yubei District Hospital of Traditional Chinese Medicine, Chongqing, China; ^5^China Academy of Chinese Medical Sciences, Beijing, China

## Abstract

**Objective:**

Moxibustion is a complementary therapy that has been used for thousands of years. Burning moxa produces smoke and inhalable particulates. Recent research has indicated that smoke inhalation is associated with negative lung effects. This study aimed to evaluate the lung function of rats after moxa smoke exposure at different concentrations.

**Methods:**

Using a randomised block experiment design, 28 male Wistar rats were randomly divided into three moxa smoke groups (opacity) (n=7): low concentration (27.45 mg/m^3^), medium concentration (168.76 mg/m^3^), and high concentration (384.67 mg/m^3^) with a control group. Rats in the moxa smoke groups were exposed in an automatic dynamic exposure device separately with different concentrations for 20 min/d, 6d/week, for 24 weeks. Rats in the control group were exposed in the same space without moxa smoke. Lung function was evaluated by the AniRes 2005 animal pulmonary function analysing system. Statistical Product and Service Solutions 18.0 software was used for data analysis.

**Results:**

In the study, no deaths were found in any group. There was no difference of forced expiratory volume in one second/forced vital capacity percentage (FEV1/FVC%), inspiratory resistance (Ri), and expiratory resistance (Re) among each group after 24 weeks of moxa smoke exposure (P>0.05). Compared with the control group (0.33 ml/cmH_2_0), dynamic compliance (Cdyn) was reduced in the medium (0.29 ml/cmH_2_0) and high (0.25 ml/cmH_2_0) concentration groups (P<0.05); however, Cdyn in the low concentration group (0.29 ml/cmH_2_0) was not significantly affected.

**Conclusion:**

Moxa smoke exposure at low concentrations did not affect the rat's lung function. Moxa smoke of medium and high concentrations destroyed the lung function represented by decreased Cdyn. However, moxa smoke of low concentrations (27.45 mg/m^3^) is much higher than the concentration in a regular moxibustion clinic (3.54 mg/m^3^). Moxa smoke at higher concentrations might destroy the lung function. The safety evaluation of moxa smoke requires further research.

## 1. Introduction

Moxibustion, an important component of clinical therapeutics of Traditional Chinese Medicine, has reliable efficacy and the unique advantages of warming meridians and stimulating acupuncture points. With its extensive application scope and convenient operation, moxibustion is used widely in China and other Asian countries to treat diseases. In the process of moxibustion, moxa smoke and heat are generated. Heat has been proven to play a role by thermal stimulation [1]. Recent studies have shown that moxa smoke also had anti-inflammatory, antitumour, and antibacterial effects [2–4]. Ancient Chinese books contain records of the use of moxa smoke in the treatment of the common cold, headache, cough, and toothache [5]. Additionally, the methanol extract of moxa smoke has the functions of antioxidation and eliminating free radicals [6].

However, the effects of moxa smoke have become controversial. In recent years, smoke-related safety has been a topic of concern, and studies showed that cigarette smoke inhalation induced pulmonary inflammation [7, 8], pulmonary granulomas, pulmonary alveolar epithelial hyperplasia [9, 10], and many other toxic effects. Since then, many countries have banned smoking in public places. With its similar route of exposure, moxa smoke has become the focus of several studies.

The aim of the present study was to evaluate lung function in rats with long-term moxa smoke exposure. Simulating the exposure mode of practitioners in clinics, rats were repeatedly exposed to moxa smoke in an experimental device. Three groups of male Wistar rats (n=7/group) were exposed to three different concentrations and one control group (n=7/group) was exposed to clean air, 20 min/d, 6d/week for 24 weeks. Three different concentrations (27.45 mg/m^3^, 168.76 mg/m^3^, and 384.67 mg/m^3^) were equivalent to 1/1000, 1/20, and 1/10 LD50 (the lethal dose of 50%), respectively, which was obtained from the pretest acute toxicity study. The rats' lung function was tested by the AniRes2005 lung function system (Bestlab, AniRes2005, version 2.0, China).

## 2. Material and Methods

### 2.1. Animal Preparation

Male Wistar rats of 3 weeks of age and weighting 50-70 g were obtained from the Experimental Animal Centre of the Academy of Military Medical Sciences in Beijing, China (certificate of quality no: SCXK-2012-0001). Rats were kept in chambers in a barrier system room under controlled environment conditions at the temperature (TEMP) of 22±0.5°C, relative humidity (RH) of 30 to 70% and a 12-h light-dark cycle. Food and water were supplied ad libitum.

The animal protocols were approved by the Ethics Committee on Animal Research of the Academy of Military Medical Sciences in Beijing, China. All procedures for animal experiments were conducted in accordance with the World Health Organisation's International Guiding Principles for Biomedical Research Involving Animals.

### 2.2. Moxa Smoke Exposure

Moxa smoke was produced by burning moxa sticks (three-year-old pure moxa, 180 mm×200 mm×10, Nanyang Hanyi Moxa Co., Ltd., Henan, China), which are widely used by the Chinese population. Moxa sticks were encased in mugwort floss, which was made of dried mugwort leaves. The moxa sticks were produced with a 3:1 ratio, which means that 3 kg of dried mugwort leaves were processed into 1 kg of moxa floss. Moxa sticks were conditioned in a sealed room at a temperature of 20 to 25°C for 7 to 21 days before burning.

Moxa smoke was delivered into the exposure cabin in an exposure device (HRH-CSED-A, Beijing Huironghe Technology Co., Ltd., Beijing, China), which was mimicking the whole-body mode of practitioners exposed in clinics. The device was an automatic dynamic exposure device. The concentrations of oxygen (O_2_) and moxa smoke were regulated by environmental monitoring and a controlling subsystem. Rats were exposed to moxa smoke in an exposure chamber (75 cm×50 cm×50 cm) at a target value, which was displayed as shading rate (SR: x %) (L=75 cm). Moxa smoke produced by burning mugwort contained several volatile oils, including eucalyptol (29.08%), camphene (11.23%), borneol (9.25%), camphor (7.97%), and p-Menth-1-en-4-ol (3.84%), which was analysed by gas chromatography-headspace spectrometry [11].

Rats were quarantined and acclimatised for 1 week before the start of the experiment. Twenty-eight Wistar rats were randomly divided into 4 groups (n=7/group) using the random number table method. Three groups of male Wistar rats were exposed to three different concentrations (10%, 40%, and 70%), and one control group (C) was exposed to clean air for 20 min/day for 144 days. Particulate matter less than 10 microns (PM10) were measured by the light-scattering digital dust tester (DT, Beijing BINTA Green Technology Co., Ltd.) in 10% and 40%, and they were 27.45 mg/m^3^ and 168.76 mg/m^3^. PM10 in 70% was calculated by MATLAB analysis, and it was 384.67 mg/m^3^. The daily moxa smoke exposure order was as follows: the control group, 27.45 mg/m^3^ group (L), 168.76 mg/m^3^ (M), and 384.67 mg/m^3^ group (H).

### 2.3. Lung Function Measurement

Twenty-four hours after the final exposure, lung function was assessed. Rats were weighed and then deeply anaesthetised by an intraperitoneal injection of pentobarbital (100 mg/kg). According to the manufacturer's instructions of the AniRes2005 lung function system (Bestlab version 2.0, AniRes2005, China), each rat was connected to a computer-controlled small-animal ventilator via a tracheal cannula. They were placed supine inside a plexiglass whole-body plethysmograph. The pressure changes in the plethysmographic chamber were measured through a port in the connecting tube with a pressure transducer. Forced expiratory volume in 1 second/forced vital capacity percentage (FEV1/FVC%), inspiratory resistance (Ri), expiratory resistance (Re), and dynamic compliance (Cdyn) were determined as changes of airway function.

### 2.4. Microscopic Examination

After the lung function measurement, each rat was subjected to full necropsy. The middle lobe of the right lung was preserved in 10% neutral buffered formalin, desiccated, and embedded in paraffin. Five-micrometre-thick sections were cut and were stained with the standard haematoxylin and eosin (HE) method. The histopathological observations were conducted under a light microscope (Nikon).

### 2.5. Data Analysis

SAS 8.2 statistical software was used for data analysis. The data of body weights, FEV1/FVC%, Ri, Re, and Cdyn was not distributed normally, and a nonparametric test was used to compare data from different groups. The data was expressed as median and interquartile range (M (q1-q3)), and multiple comparisons were conducted using analysis of variance (ANOVA) based on rank. Deviation p<0.05 was considered to be statistically significant.

## 3. Results

### 3.1. Mortality and General Condition Observation

During 24-week exposure, no death occurred in any of the moxa smoke-exposed groups. Rats had normal diet and stools, and no abnormal appearance and behaviour were found.

### 3.2. Body Weight

**Figure 1:**
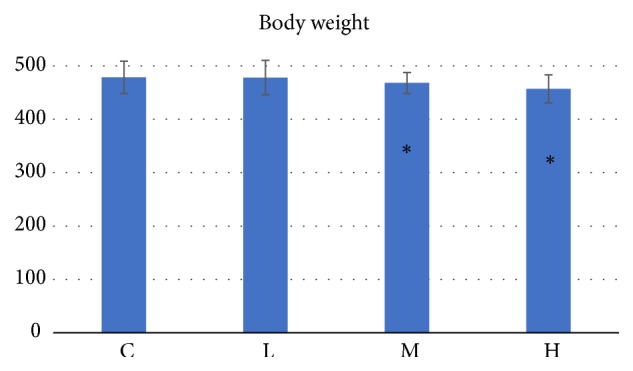
Body weight after 24-week moxa smoke exposure. C: the control group, which were exposed to clean air. L: the low concentration group, which were exposed to 27.45 mg/m^3^ moxa smoke. M: the medium concentration group, which were exposed to 168.76 mg/m^3^ moxa smoke. H: the high concentration group, which were exposed to 384.67 mg/m^3^ moxa smoke. Sig^a^, the significance for data among the groups. “ ^*∗*^” p<0.05 versus C group.

After 24-week moxa smoke exposure, the rats in L group (477.9±32.3g) showed no significant changes in body weight compared with the rats in C group (478.6±30.2g). However, the body weight of the rats in the M group (468.1±19.7g) and H group (457.1±26.3g) decreased, respectively, compared with those of the C group (Figure 1). The changes of rats and body weight reflected the growth and development of rats, while the food and water intake were not measured in moxa-exposed rats. In this study, the reduction of body weight in the M and H groups might show toxic reactions.

### 3.3. Lung Function Measurement

FEV1/FVC%, Ri, Re, and Cdyn were analysed and exported by the AniRes2005 lung function system (Table 1). There was no significant difference in FEV1/FVC%, Ri, and Re among different groups, while the Cdyn in the M (0.29) and H (0.25) groups was lower than that in the control group (0.33). The difference was statistically significant (P<0.05). However, Cdyn in the L group showed no significant difference as compared with the control group.

### 3.4. Microscopic Examination

**Figure 2:**
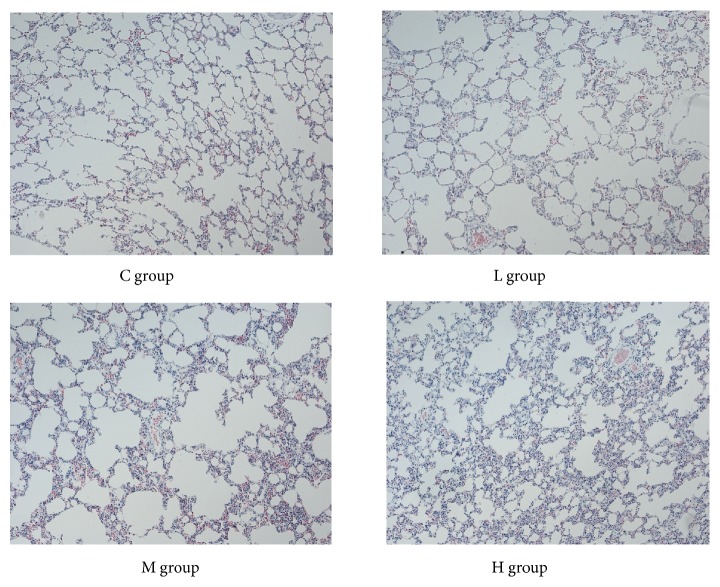
Microscopic observation of lung pathology (HE stain, ×10).

**Figure 3:**
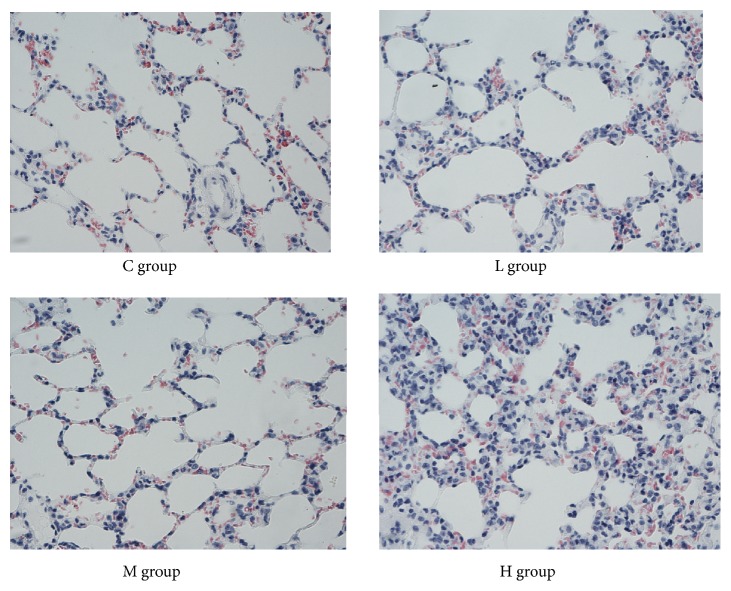
Microscopic observation of lung pathology (HE stain, ×40).

The structure of alveolar cells in the L and M groups showed no obvious changes. However, the alveolar septa of rats in the H group were thicker than those in the C group. Histological changes, such as inflammatory granulocyte infiltration, focal haemorrhages, and alveolar cavity expansion, could be found in the lungs of rats in the H group (Figures 2 and 3).

## 4. Discussion

In this study, we evaluated the lung function of rats after 24-week repeated moxa smoke exposure at three different concentrations, which were designed by toxicological methods. This study found that moxa smoke exposure at low concentrations (27.45 mg/m^3^) did not reduce the lung function of the rats. Moxa smoke exposure at medium concentrations (168.76 mg/m^3^) and high concentration (384.67mg/m^3^) decreased lung dynamic compliance. The safety of moxa smoke has received close attention. The question of whether the moxa smoke is harmless or not has become the key to restrict the use of moxibustion.

The particulates in the air of moxibustion treatment rooms are part of moxa smoke, which are produced by burning mugwort, a material different from other burned materials. At present, the other studies of inhalable particulate matter are involved in air pollution, cigarette smoke, mosquito coil smoke, etc. Inhalable particulate in the environment has become a major hazard to human health, especially the respiratory system. Jedrychowski [12] assessed the effect of low concentrations of ambient air pollution on lung function growth in preadolescent children and found that air pollution in the residence area may be a part of the causal chain of reactions leading to retardation in pulmonary function growth. Exposure to pollutional haze, the carrier of air pollutants such as particulate matter and nitrogen dioxide (NO_2_) has been linked to lung and cardiovascular disease [13]. Cigarette smoke and mosquito coil smoke were found to be bad for lungs and lung function. Two studies [14, 15] reported that current exposure to environmental tobacco smoke in healthy male adolescents and steroid-naive preschool children were associated with lung function impairment of the effects of maternal smoking. Animal experiments found that in utero tobacco smoke exposure significantly affected growth and development in mice and decreased antioxidant defences concomitantly with impaired lung function, which was associated with high-mobility group box 1 protein (HMGB-1) expression [16, 17]. These impacts might partially explain the susceptibility of infants born to smoking mothers to early respiratory disease and chronic respiratory disease as adults. Another study demonstrated that current smoking was significantly relevant to symptoms of asthma, such as having recent wheezing and recent exercise-induced wheezing in Korean adolescent populations [18]. What is more, a social survey [19] reported that initiation of cigarette smoking and exposure to second-hand smoke in adolescence led to increased respiratory symptoms and reduction of pulmonary function test values. In addition to cigarette smoke, mosquito coil smoke also causes damage to the lungs. Environmental exposure may play a role in lung cancer risk. In a study in Taiwan, in which questionnaires were administered to 147 primary lung cancer patients and 400 potential controls, the risk of lung cancer was significantly higher in frequent burners of mosquito coils [20]. Another study of three cases reported that small cell lung cancer was likely to be the result of exposure to mosquito coils in a Shanghai pulmonary hospital [21]. Contact with mosquito coil smoke could expose individuals to a level of S-2 that may increase the risk of lung cancer. Additionally, an animal study demonstrated that exposure to allethrin-based mosquito coil smoke for prolonged periods of time could lead to oxidative stress [22].

Although it was suggested that the inhalable particulate matters were harmful for lungs, low and middle concentrations could not affect lung function. Most of the studies on smoke have revolved around lung diseases and function because it is the most direct impact after inhalation. Pulmonary function tests can be used to analyse forced expiratory volume in 1 second/forced vital capacity ratio (FEV1/FVC%), inspiratory resistance (Ri), expiratory resistance (Re), and lung compliance (Cdyn). FEV1/FVC% plays an important role in the judgement of pneumoconiosis, pulmonary function ventilation and obstructive disorders [23]. Airway resistance is a good indication of airway obstruction and helps to determine whether the cause of pulmonary dysfunction is derived from the airways or not [24]. Bronchial asthma, emphysema, and obstructive ventilatory dysfunction all contribute to increased airway resistance [25, 26]. Cdyn refers to changes in lung volume caused by unit pressure without interruption of airflow during the respiratory cycle, and it is affected by both lung tissue elasticity and airway resistance. Reduction in Cdyn is associated with restrictive lung disease, alveolar filling disease, acute respiratory distress syndrome, and emphysema [27, 28]. The decrease of lung dynamic compliance might be caused by inflammatory granulocyte infiltration and alveolar cavity expansion. Current smoking was further associated with lower pulmonary function. However, it was recorded that moxibustion had a positive effect on pulmonary fibrosis with the produce of moxa smoke [29].

Earlier research of our group proved that a fixed concentration of moxa smoke was safe [11]. Moreover, John Wheeler compared moxa smoke with cigarette smoke and found levels of only two volatiles produced were equivalent or greater than the safe exposure levels [30]. A study in Japan showed that quantities of harmful substances released upon combustion of moxa during normal clinical therapy in Japan fell below national safety standards [31]. Korean researchers found that the CO/NOx/VOC level of moxa smoke remained within safe limits [32]. The effect of moxa smoke is similar to that of aromatherapy, according to several studies [33, 34]. Moxa smoke, the combustion product of mugwort, is complicated. In the moxa smoke 61 peaks, which were separated from the combustion product of mugwort, were characterised by GC-MS, and among them, 26 components were identified. Most of the combustion products are essence and aroma, such as benzaldehyde, phenol, o-cresol, p-cresol, caryophyllene, and caryophyllin [35]. A few of them have some toxicity at high concentrations, and they are harmless to humans at low concentrations, such as phenol and catechol, which are often used as disinfectants [36]. A study [1] showed that moxa smoke increases PM10 concentration, but the oxidative capacity of PM10 in the moxibustion room was much lower than that at control sites with the same particulate burden. Huang Jian [37, 38] contrasted PM2.5 concentration produced by three different ratios of moxa in simulated moxibustion clinics and found that the oxidative DNA damage induced by individual PM2.5 following moxibustion was lower than that reported in other environments. Similar studies [39, 40] suggested that PM10 and gaseous pollutants might not be as injurious to human health as generally assumed.

In the present study, three moxa smoke concentrations (27.45 mg/m^3^, 168.76 mg/m^3^, and 384.67 mg/m^3^, respectively) were equivalent to 1/1000, 1/20, 1/10 LD50 respectively, which was obtained from the pretest acute toxicity study. Moxa smoke is different from other inhalable particulates. Moxa smoke is inevitable in the course of moxibustion treatment. This study was a systematic moxa smoke toxicology experiment, and the safety of moxa smoke requires longer term toxicity testing. Considering the feasibility of the experiment, rats' exposure period (20 min) was less than the time of acute toxicology experiments. An acute toxicology study about moxa smoke suggested that exposure to 10% moxibustion-derived burning products was under the critical threshold for rats' safety [41]. However, there were some limits. Although better control of the parameters was established and the moxa smoke concentration could be displayed in real time and automatically controlled, the concentrations of CO and SO_2_ emissions were not monitored. Due to time and space restrictions of the automatic dynamic exposure device, only male rats were chosen for the study. In future studies, the concentrations of CO, CO_2_, SO_2_, NO, VOC and inhalable particulate matter should be monitored during the experiment. Moreover, female and male rats should be listed as experimental animals for further research. PM10 concentration in a regular moxibustion clinic was 3.54 mg/m^3^ [42]. Low concentration moxa smoke of 27.45 mg/m^3^ is much higher than the concentration in a regular moxibustion clinic. Clinical observation and epidemiological investigation using larger samples should be added.

In conclusion, moxa smoke at low concentrations did not affect the rat's lung function in 24 weeks. Moxa smoke at higher concentrations might destroy the lung function. In clinical settings, moxibustion treatment is relatively safe; however, further safety evaluation of moxa smoke is needed.

## Figures and Tables

**Table 1:** Lung function FEV1/FVC%, Ri, Re and Cydn after moxa smoke exposure.

group	FEV1/FVC%	Cdyn (ml/cmH_2_O)	Re (cmH_2_O•s/ml)	Ri (cmH_2_O•s/ml)
	(M(q1-q3))	(M(q1-q3))	($\overset{-}{x}\pm s$)	($\overset{-}{x}\pm s$)
C	21.88 (16.10-23.33)	0.33 (0.32-0.41)	0.41±0.04	0.34±0.03
L	19.08 (18.74-22.37)	0.29 (0.27-0.36)	0.43±0.10	0.30±0.05
M	23.19 (20.15-24.48)	0.29 (0.25-0.32)^*∗*^	0.38±0.09	0.33±0.05
H	22.56 (22.00-24.47)	0.25 (0.23-0.33)^*∗*^	0.36±0.08	0.32±0.03

Data was shown as median and interquartile range (M (q1-q3)).

C: the control group, which were exposed to clean air. L: the low concentration group, which were exposed to 27.45 mg/m^3^ moxa smoke. M: the medium concentration group, which were exposed to 168.76 mg/m^3^ moxa smoke. H: the high concentration group, which were exposed to 384.67 mg/m^3^ moxa smoke.

Sig^a^, the significance for data among the groups. “ ^*∗*^” p<0.05 vs C group.

## Data Availability

The data used to support the findings of this study are available from the corresponding author upon request.
